# The kingdom’s emerging elderly care landscape: insights for the future of elder care in Saudi Arabia: a systematic review

**DOI:** 10.3389/fpubh.2026.1825664

**Published:** 2026-06-24

**Authors:** Turki Alsaeed, Tracy Washington, Bo Xia

**Affiliations:** School of Architecture and Built Environment, Faculty of Engineering, Queensland University of Technology, Brisbane, QLD, Australia

**Keywords:** aging challenges, elderly care, elderly care reforms, population aging, Saudi Arabia

## Abstract

**Background:**

Saudi Arabia is undergoing a major demographic transition, with the proportion of older adults projected to increase from 5% in 2015 to over 20% by 2050. Although improvements in healthcare have extended life expectancy, the rapid growth of the elderly population is placing increasing pressure on the country’s health and social care systems. Persistent challenges—including shortages in the geriatric workforce, heavy reliance on migrant caregivers, cultural reluctance toward institutional care, and disparities in rural service provision—limit the system’s ability to respond effectively. While Vision 2030 has introduced reforms promoting integrated, modernized, and digitally enabled elder care, the evidence base specific to aging in Saudi Arabia remains limited and fragmented. This systematic review aims to consolidate existing evidence, identify critical gaps, and evaluate the alignment of current initiatives with Saudi Arabia’s demographic trajectory.

**Methods:**

This PRISMA-guided systematic review consolidates existing literature to assess the current state of elderly care in Saudi Arabia, identify system gaps, and evaluate how ongoing initiatives align with the nation’s demographic trajectory. Sources published in English or Arabic between 1995 and 2025 were retrieved from major academic databases and grey literature. Studies were screened for relevance and subsequently analyzed using thematic synthesis.

**Results:**

The review indicates that Saudi Arabia’s elderly care system is evolving but remains under strain. Expansion of long-term care services, hospital-based geriatric programs, and policy alignment with Vision 2030 reflect measurable progress. However, several barriers persist, including a growing chronic disease burden, rising healthcare costs, digital illiteracy among older adults and some providers, and continued dependence on foreign labor in caregiving roles.

**Conclusion:**

This systematic review finds that Saudi Arabia’s elderly care system is evolving but faces persistent structural, workforce, and cultural challenges that require urgent attention. Strategic, technology-supported, and workforce-focused reforms are essential to meet the demands of a rapidly aging population. Significant gaps in data and research on aging in the country remain, underscoring the need for further evidence to support informed policy development and long-term planning.

## 1 Introduction

Saudi Arabia is undergoing a period of substantial demographic changes (1). In 2015, only 5% of the total population was aged, but this number is anticipated to quadruple to 20.9% by 2050 (2). The Elderly Care Department at the Ministry of Human Resources and Social Development (HRSD) highlights this surge, noting an estimated 1.5 million elderly individuals in the Kingdom, a significant rise that underlines an urgent need for consideration of this demographic (3). This is contributed to the advancements in the diagnosis and treatment of diseases that reduce the mortality rate, resulting in a growing population of senior citizens (4, 5). The Custodian of the Two Holy Mosques and His Royal Highness the Crown Prince have consistently issued directives to enhance citizen services, making the prioritization of the care for older residents a national imperative (3).

The elderly care sector in Saudi Arabia encompasses residential care homes, home-based care, and community programs, with the Ministry of Health (MOH) and Ministry of Human Resources and Social Development (HRSD) leading service provision. The strategic vision for elderly care developed by the Ministry aims at increasing awareness regarding the requirements of older adults, drawing primarily on best practices from Gulf Cooperation Council (GCC) countries whose close cultural and religious values closely reflect those of Saudi Arabia, while also referencing international models from nations such as Sweden, Australia, the United States, the United Kingdom, Germany, and the Netherlands (3). While drawing insights from these models, Saudi Arabia is customizing its strategy to reflect its distinct values and culture, ensuring that new services integrate, rather than isolate, the elderly (3). HRSD’s twelve senior residential care homes, which also provide financial and in-kind support to the needy elderly, reflect efforts to address these needs (6).

Currently, the Saudi government is actively investing in specialized elderly care facilities to enhance the quality of life for its older population (3). Under the National Transformation Program (NTP), the ministry is involved in establishing five model units designed to serve the health and psychological needs of older individuals, complete with physical therapy services and health clubs (7). Alongside, the Ministry supports the establishment of 13 specialized non-profit associations focused on elderly services, aiming to achieve nationwide coverage across Saudi Arabia. Furthermore, in collaboration with the Family Affairs Council, the Ministry is involved in drafting the Elderly Rights Law, a legislative effort aimed at safeguarding the elderly from abuse, securing their rights, and ensuring the provision of optimal, needs-based services (7). It also prioritizes enhancing elderly care through initiatives like the Seha Virtual Hospital and the “Taqdeer” service by the Civil Affairs Agency, aiming to improve accessibility and quality of life (7). Collectively, these efforts reflect a national commitment to developing an integrated, rights-based system of elderly care that aligns with Saudi Arabia’s broader social transformation goals.

However, Saudi Arabia’s elderly care sector also faces various challenges, including cultural resistance to institutional care, limited geriatric training, and rural access disparities, further exacerbated by a reliance on migrant workers (8). The aging population in Saudi Arabia is also expected to experience a significant burden of non-communicable diseases (NCDs), including diabetes, stroke, depression, cardiovascular diseases, osteoarthritis, chronic kidney disease, cancer, and dementia (9). These conditions will require expanded geriatric specialist services, long-term care facilities, rehabilitation programs, home-based care, and community support networks to meet the complex care needs of older adults (4, 8). Additionally, despite growing attention to aging populations globally, there remains a noticeable lack of research and published data that specifically address the context of Saudi Arabia’s elderly care sector. This knowledge gap hinders a comprehensive understanding of the sector’s unique challenges, needs, and opportunities within the Kingdom. By identifying and analyzing the available evidence, this research seeks to highlight these gaps and provide a foundation for future research and policy development by summarizing the current knowledge and information, providing an informative and comprehensive picture of elderly care in Saudi Arabia.

This study aims to provide a comprehensive, evidence-based synthesis of the current state of elderly care in Saudi Arabia, identify critical gaps in services and research, and offer actionable insights for policymakers, scholars, and practitioners. For consistency, this study primarily uses the term “elderly,” reflecting its common usage in the Saudi Arabian context. Where relevant, terms such as older, geriatric, and senior citizens/adults are used interchangeably and refer to the same population group.

## 2 Methods

This study employed a PRISMA-guided systematic review methodology, covering literature published between 1995 and 2025, to ensure a thorough search and evaluation of studies related to the elderly care industry in Saudi Arabia (10, 11). The review drew on reputable scholarly sources, official government resources (the Saudi Ministry of Health and the General Authority of Statistics), media articles, official statements, and in-depth knowledge obtained through a podcast interview with Ms. Ibtisam Al-Humaizi, the Director General of the Elderly Care Department, HRSD. This multi-source approach ensured a thoughtful representation of the current situation, developments, and trajectory of elderly care in Saudi Arabia. The review involved searching, screening, and analyzing sources based on inclusion criteria, and covered the following thematic areas: the historical development of elderly care, market structure and service categories, modernization efforts, governance, resident demographics and health profiles, human resources, and financing models.

### 2.1 Search study

The initial step involved searching for literature that provided general data on the elderly care sector and its evolution. Key words containing the groupings of (elderly OR aged OR older OR aging OR senior OR geriatric) AND (care OR service OR healthcare) OR (long term care, nursing home, assisted living, residential facilities, social homes) were used. For specificity, the literature available on Saudi Arabia was screened. Articles published between 1995 and 2025, written in English or Arabic, were investigated in the Scopus, Wiley Online, PubMed, ResearchGate, Consensus, Elicit, and Google Scholar databases or search engines. Grey literature was also accessed; grey literature is defined as articles that were either unpublished or published in non-commercial forms (policy statements, reports, working papers, government documents, issues papers, etc.) (12). This review is based on and examined both grey literature and scholarly, peer-reviewed literature, referred to as traditional literature, which focuses on the elderly care system in Saudi Arabia.

### 2.2 Screening

**Figure 1:**
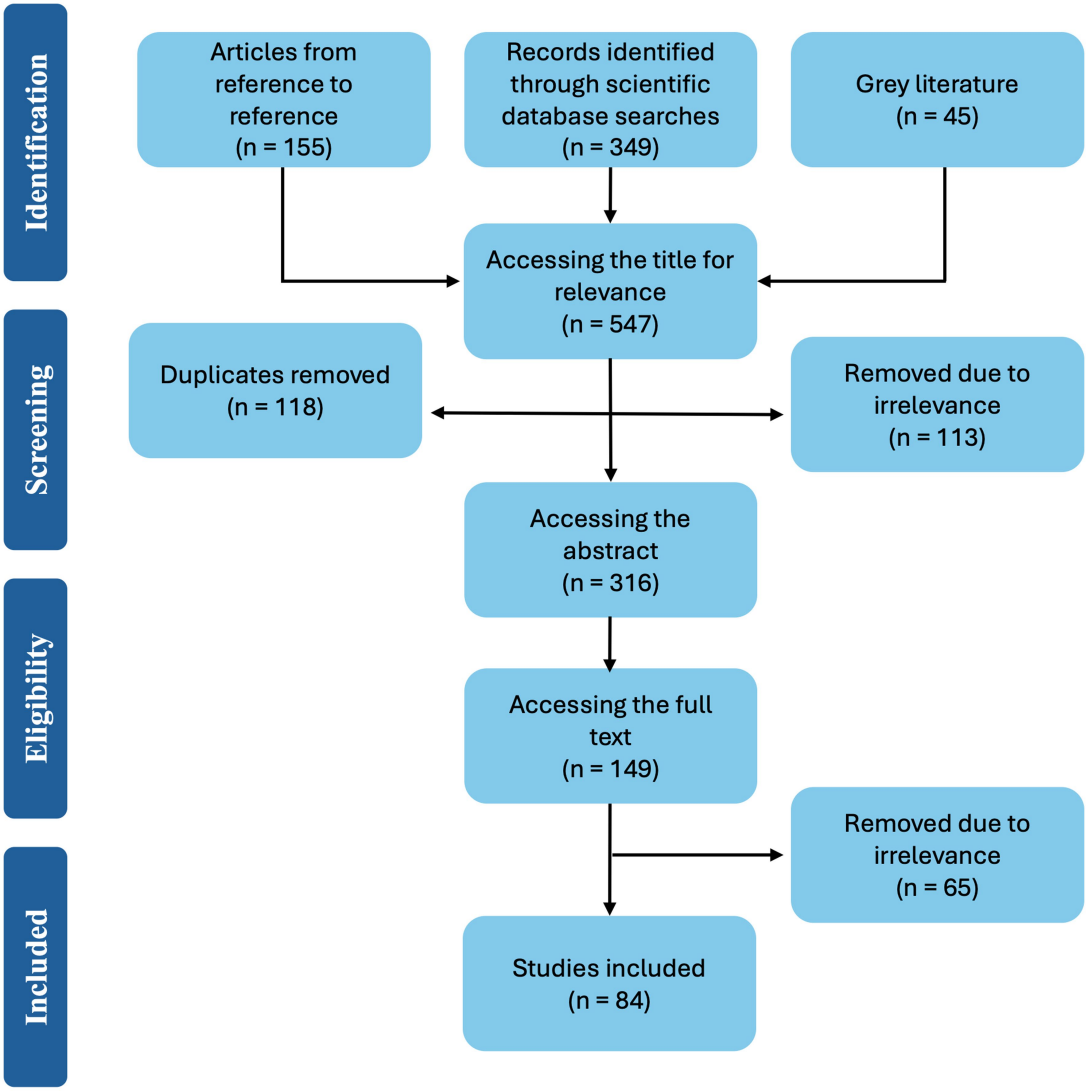
Flow chart outlining the steps involved in selecting studies for the systematic review.

The result of this search was access to 555 articles, including grey literature. The search has helped identify all studies systematically, based on their relevance to the research objectives, by examining the title, abstract, and full paper. Lastly, this study included 84 articles. The study selection steps are illustrated in the flow chart in Figure 1.

### 2.3 Analysis

A thematic synthesis approach was employed to integrate the findings, with qualitative data analyzed for recurring patterns and quantitative data (e.g., demographic statistics) summarized descriptively. The review prioritized peer-reviewed studies and official reports from organizations and the media for contemporary developments. Given the scarcity of data specific to elderly care, triangulation was performed by cross-verifying themes and trends across different data types (academic, governmental, and media sources) to enhance the credibility and consistency of interpretations. This multi-source approach strengthened the robustness of the analysis.

## 3 Results

### 3.1 Elderly care system: an overview

Elderly care, commonly termed as aged care, encompasses long-term and social support services designed for elderly individuals who require ongoing health and nursing support due to chronic disabilities and a diminished ability to function independently (13). These services, including residential care, home-based care, respite services, transitional care, and hybrid care, vary in organization, funding, and provision across countries (14). Older adults often face increased socio-economic precarity due to systemic discrimination and the disproportionate effects of health crises (15). Quality senior care extends beyond physical requirements; it emphasizes a person-centered approach that prioritizes social and emotional well-being (16, 17). In a progressively aging global demographic, nearly 15% of the world’s population cope with disabilities, largely within developing nations (15). The world’s older populations are expected to increase to approximately two billion people by 2050 (18).

#### 3.1.1 Historical and contemporary approaches to elderly care

Globally, the aged care industry has evolved through four phases (19). The Exploratory Phase (1986–1995) involved a disintegrated analysis of senior care, primarily focusing on the efficacy and immediate influencing factors of care facilities. Early insights from Saudi Arabia highlighted a growing awareness of costs and staffing (19–22). This was followed by the Constructive Phase (1996–2004), which represented a major transition aimed at improving long-term care and elevating the quality of life for seniors, while promoting the idea of coordinated medical and nursing services (22, 23). The Improvement Phase (2005–2013) further advanced this understanding by stressing the importance of proactive and preventive care (24, 25), as well as the vital role of in-home support, while expanding the focus to incorporate palliative and end-of-life care to enhance overall well-being. Finally, the Synergistic Development Phase (2014–present) is marked by diverse and intelligent methodologies, where research harnesses emerging technologies such as big data and Artificial Intelligence (AI) for real-time tracking and enhanced service delivery (26, 27). This is coupled with the effort to tackle public health emergencies, reflecting an ongoing movement toward technologically integrated and comprehensive solutions for elderly care.

In the Middle East, traditional extended-family elderly care is evolving under the weight of urbanization and shifting social structures (28). Studies from Lebanon, Iran, Turkey, Israel, the Gulf Cooperation Council, and other Arab countries document a steady shift from multigenerational households to more nuclear and hybrid arrangements (living alone and away rather than co-residing within extended family) (29). In urban environments, the physical separation between family members has reduced the practicality of direct care across generations (30). In Saudi Arabia, older individuals were traditionally cared for within extended family households, reflecting Quranic principles of filial piety (Quran 17:23–24). However, longstanding norms of filial duty (the expectation that children care for their aging parents) are transitioning to configurations that integrate both informal family assistance and formal care services (30). This shift is further shaped by broader socio-economic and policy-driven changes, including increased female participation in the formal workforce as part of national development initiatives. While this transition reflects evolving gender roles, it also responds to structural and economic demands. As more women engage in paid employment, the continued reliance on them as primary caregivers intensifies caregiving pressures, particularly within smaller family networks (4, 31). Collectively, these factors illustrate how urbanization, economic transformation, and changing social structures are reshaping traditional elder care practices in the region.

#### 3.1.2 Timeline and milestones in Saudi elderly care

Saudi Arabian elderly care emerged from a long-established, family-centered practice steeped in cultural and religious norms. In their 2022 study, the researchers state the clash of narratives, explaining that Saudis oppose the institutionalization of older adults in long-term care facilities based on artistic grounds, particularly the religious requirement that one should be kind to one’s elderly parents (32). The evolution of elderly care in Saudi Arabia reflects a gradual shift from exclusively family-based care to more diverse models that incorporate institutional and technology-assisted services (Table 1). While the pre-2000 period was dominated by informal, multigenerational family support, the 2000–2010 decade marked a transitional phase characterized by the emergence of social care homes and hospital-based services. In the most recent period, a hybrid system has developed, combining home-based, institutional, and technology-supported care, though family care remains culturally dominant.

**Table 1:** Historical mapping.

Time period	Care type model	Key features	Cultural context	Socioeconomic drivers
Pre-2000	Family-based (33)	Multigenerational households, informal care by women (33)	Strong religious/cultural norms; institutional care stigmatized (33)	Pre-oil boom tribal structures; limited urbanization; no policy framework (35)
2000–2010	Transitional (35)	Emergence of social care homes, hospital-based long-term care (35)	Urbanization, industrialization, and family structure under pressure (35)	Oil-driven urbanization; rising female employment; nuclear family formation (4, 31)
2010–present	Mixed/Hybrid (34)	Social care homes, home-based care, technology (ambient assisted living) (38)	Gradual acceptance of institutional care, but family care is still dominant (4, 34)	Vision 2030 policy impetus; 2022 Elderly Rights Law; digital health investment; PPP frameworks (36, 37)

The evolution of Saudi elderly care cannot be understood without situating it within the Kingdom’s sweeping socioeconomic transformation. Prior to the oil boom of the 1970s, Saudi society was organized around tribal and extended-family networks in which the care of elderly relatives was an intrinsic moral and religious duty. The Quranic injunction in Surah Al-Isra (17:23–24)—commanding filial kindness and forbidding even the mildest expression of impatience toward aging parents—shaped an overwhelmingly informal care system in which institutional alternatives were not merely absent but actively stigmatized (32, 33). Elderly individuals who could not be cared for by family were perceived as having been abandoned, and this stigma attached equally to families who placed relatives in care homes and to the institutions themselves (33, 34).

The oil revenues of the 1970s–1980s triggered urbanization on a massive scale, pulling younger Saudis into cities and dissolving the multigenerational compound households that had sustained informal care. For the first time, geographic distance between adult children and aging parents became common, placing structural pressure on the filial duty norm without yet producing a policy or institutional response (4, 35). The first tentative government acknowledgment of elder care as a formal social responsibility emerged in this period, primarily through the Social Welfare Law administered by the Ministry of Social Affairs (predecessor to today’s HRSD). Social care homes were established not as preferred alternatives to family care but as safety nets for elderly individuals who were wholly without family—widows, divorced women, and childless men (33).

The 2000–2010 decade represented a decisive transitional decade. Rapid urbanization, rising female labor force participation, and the nuclearization of family units combined to erode the practical foundations of exclusively family-based care (4, 31). Mufti (2002) made an early case for community and hospital-based long-term care facilities as a necessity rather than a luxury, a call that was ahead of its time but that resonated increasingly with policymakers over the following two decades (33). Hospital-based geriatric programs began to appear in larger urban centers such as Riyadh, Jeddah, and Dammam, and a small number of private nursing facilities emerged, catering primarily to affluent families seeking respite or specialist dementia care. Crucially, however, the stigma around institutional care had not dissolved—it had merely softened among urban, educated, higher-income segments of the population (34).

The 2010s saw a qualitative shift driven by two converging forces: demographic urgency and policy ambition. The publication of demographic projections showing that Saudi Arabia’s elderly population would grow from approximately 5% to over 20% of total population by 2050 elevated elder care from a peripheral social welfare concern to a mainstream healthcare policy priority (2). The launch of Vision 2030 in 2016 institutionalized this elevation, embedding elder care reform within the Healthcare Transformation Program (HTP) and aligning it with national goals of privatization, digitalization, and workforce Saudization (36). The 2022 Elderly Rights and Care Law marked the most substantive legal milestone since the Social Welfare Law, establishing enforceable rights, a care responsibility hierarchy, and criminal penalties for financial abuse—a significant normative departure from purely discretionary welfare provision (7, 37).

In terms of residual stigma, evidence suggests that attitudes are stratified by education, geography, and income. Rural and less-educated populations retain stronger taboos against formal care placement, consistent with findings from comparable MENA societies (28, 29). Among educated urban populations, the shift is more pronounced: Karlin et al. (34) documented a growing acceptance of institutional care among contemporary Saudi older adults themselves, many of whom expressed preferences for services that preserved independence and social engagement. This generational and geographic divergence has significant planning implications: care infrastructure must be differentiated rather than uniform, and community-based and home care models are likely to achieve higher uptake than residential facilities for the foreseeable future (38, 39).

#### 3.1.3 Market structure and scope of elderly care services in Saudi Arabia

The deeply rooted tradition of caring for older individuals within the family unit significantly shapes both the limited uptake and the structure of formal elderly care services in Saudi Arabia. Research consistently shows that formal care programs in Saudi Arabia are infrequently utilized, with older adults primarily identifying family support as their favored form of assistance (40). Nevertheless, the growing healthcare market in Saudi Arabia increasingly reflects the rising demand for elderly-related services, driven by demographic aging and the growing burden of chronic diseases among older adults. A recent report on Saudi Arabia’s elderly healthcare market suggests that it is poised for significant growth, reflecting the country’s evolving demographic profile and healthcare priorities (41). The market, valued at approximately USD 1,012.6 million in 2024, is expected to grow substantially, reaching USD 4,542.4 million by 2033. This indicates a strong Compound Annual Growth Rate (CAGR) of 18.1% throughout the forecast period. Analysis by service type indicates that home healthcare services are expected to dominate, accounting for 43.7% of the market share in 2024. Furthermore, chronic diseases are projected to be the leading segment by medical condition, holding a 36.0% market share in the same year (41).

The report also highlights Saudi Arabia’s recent proactive efforts to advance its elderly healthcare sector in alignment with the objectives of Vision 2030. Introduced in 2016, Vision 2030 is leading the elderly care reforms under the Healthcare Transformation Program (HTP), which is overseen by the MOH Vision Realization Office (VRO), with privatization, workforce development, and digital innovation at the forefront (36). In November 2024, the Ministry of Health launched a government-backed telemedicine platform for elderly care, a collaborative effort with Dr. Sulaiman Al-Habib Medical Group and Saudi Telecom Company (STC), aimed at improving access in rural areas. To further strengthen professional capabilities, August 2024 marked the expansion of geriatric training programs through a partnership with global institutions such as King Faisal Specialist Hospital & Research Centre and Harvard Medical School, aimed at addressing the shortage of geriatric specialists. Technological integration is also a key focus, as demonstrated in April 2024 when Saudi German Health and MedeAnalytics introduced AI-integrated wearable devices for chronic disease management, offering real-time monitoring and remote care for conditions such as diabetes and cardiovascular diseases (41). Complementing these advancements, in January 2024, Al Habib Medical Group opened a specialized geriatric care wing in Riyadh, providing comprehensive, multidisciplinary services, including rehabilitation, chronic disease management, and dementia care, with state-of-the-art technology (41). Moreover, in September 2023, the Saudi Ministry of Health initiated a government subsidy for home healthcare services for low-income families, partnering with providers such as Care Shield Home Healthcare and Medcare Hospital, to increase the accessibility and affordability of in-home care solutions (41).

Key players in this burgeoning market include prominent providers such as Saudi German Health, Dr. Sulaiman Al-Habib Medical Group, King Faisal Specialist Hospital & Research Centre, and Al Hammadi Hospitals, among others, signaling a dynamic and competitive landscape (41). The Ministry of Human Resources and Social Development (HRSD) plays a central role in providing care and support for older adults, under the directives of the Custodian of the Two Holy Mosques and the Crown (3). The HRSD operates a limited number of government-run care homes designed primarily for older people who lack familial support or whose families are unable to provide adequate care. Ms. Ibtisam Al-Humaizi noted that there are eight such government facilities across the Kingdom, accommodating a relatively small number of individuals (around 418 in total at the time of her statement). This low occupancy rate strongly reinforces the prevailing cultural preference for family care (3).

Public-Private Partnerships (PPPs) are a key strategy in Saudi Arabia’s Vision 2030, serving as a crucial strategy to enhance and expand the economy through the development of the healthcare system, particularly in areas such as long-term care (LTC), rehabilitation, and home care (HC). These PPPs in the Saudi government are promoted by the Private Sector Participation (PSP) initiatives by the Saudi MOH, which encourages the involvement of the private sector in healthcare services (42). This strategic focus aims to significantly enhance extended care by increasing access to and the quality of services. Such developments in long-term care can help improve the quality of tertiary care hospital bed capacity and better utilize it, as well as enhance the quality of post-acute and home care services. The development of the LTC, rehabilitation, and home care sectors in Saudi Arabia is expected to bring a huge change in the healthcare sector, shifting the patients out of acute care hospitals and into special LTC and rehabilitation facilities and eventually into home-based care. This transition is crucial in alleviating pressure on the entire healthcare sector, acute care, long-term care, and rehabilitation institutions (42).

##### 3.1.3.1 Elderly care categories

Four approved types of elderly care centers operate under the Ministry’s framework: permanent residential centers, temporary shelters, day-care centers, and social clubs. The application process for establishing these centers has been digitized through the Ministry’s online platform, enabling applicants to access regulations, read the procedural guide, and submit their applications with full transparency (3). However, it is important to note that these four care categories are not unique to Saudi Arabia. Analogous categories exist in comparable healthcare systems across the MENA region and internationally. For example, Jordan and Lebanon similarly classify elder care into residential, community day-care, and home-based service streams, and the Gulf Cooperation Council (GCC) broadly shares this typological structure (28, 29). What distinguishes the Saudi instantiation of these categories is the normative and regulatory weighting applied to each. In Saudi Arabia, residential care remains a last resort, reinforced by law—the 2022 Elderly Rights and Care Law explicitly requires family care as the primary obligation. It mandates informed consent before any institutionalization (7). This legal architecture has no direct parallel in most other MENA systems, where institutional placement is governed more by market dynamics and family preference than by statutory hierarchy.

Comparatively, Saudi Arabia’s day-care center model shares characteristics with models in the United Kingdom, where day centers serve as socially integrative complements to home care rather than substitutes for it (16). The social club model, however, is more culturally distinctive: its explicit aim to involve family members alongside elderly participants reflects a Saudi-specific design logic that emphasizes intergenerational cohesion rather than age-segregated service delivery. This contrasts with Scandinavian models, which prioritize elderly autonomy and peer socialization in age-homogeneous settings (3, 43). The value of discussing these categories within a Saudi-specific frame, therefore, lies not in their novelty per se, but in documenting how globally common service types are culturally adapted and legally embedded in ways that reflect the Kingdom’s particular religious, familial, and governance values.

###### 3.1.3.1.1 Long term and post-acute Care (LTPAC)

LTPAC covers a wide range of rehabilitative and palliative care that helps to meet both medical and non-medical needs of a person with a chronic illness or disability. This type of care is provided after, or in some cases instead of, an acute care stay in a hospital setting, and it lasts a long period (44). Services offered in LTPAC are broad, with highly specialized care provided in long-term acute-care hospitals, as well as more personalized care delivered in home settings. Individuals who access LTPAC services typically have a more diverse range of conditions and more complex, often chronic, care needs than the general population. Such complexities frequently necessitate transfers between different care settings, including home, acute, post-acute, and long-term facilities. This necessitates a holistic understanding of the patient’s care management journey, which begins with discharge from acute care and continues through inpatient rehabilitation and into specialty long-term and Post-Acute Care institutions (44).

###### 3.1.3.1.2 Social care homes

Social care homes are a vital residential center offering a safe living environment to Saudi citizens aged 60 and above, who cannot fulfill their basic needs, especially those who have no proper family or social support. These facilities also accept patients and elderly people referred by hospitals who lack caregivers, provided they do not have infectious diseases or severe mental illnesses. These facilities offer comprehensive residential services that encompass social, medical, and psychological support to enhance the quality of life for residents (7). Moreover, various types of recreational, cultural, professional, and sports activities are carefully arranged to encourage social interaction and prolonged active engagement. The most significant projects include the Elderly Gathering Hall, which aims to provide a relaxing atmosphere that eliminates generational differences and facilitates the exchange of experiences, ultimately promoting intergenerational communication (7). At the same time, the new Elderly Rights and Care Law provides strong protection of elderly rights. It guarantees a high quality of life by giving special privileges and protecting their social, financial, and legal rights. One of the provisions of this act is the issuance of an Elderly Privilege Card, which grants the cardholder priority in service delivery, discounts on government services, both governmental and non-governmental, as well as simplification of procedural interactions (7).

###### 3.1.3.1.3 Day-care centers and social clubs

In daycare facilities, seniors attend during the day to engage in organized activities and then return home in the evening (45). These centers operate on a schedule similar to a standard workday, with participants attending daytime hours. They are designed to align with Saudi cultural values while addressing the desires of senior citizens (3). On the other hand, social clubs operate with greater flexibility and provide unrestricted access, allowing older individuals to drop in at any time. They are adaptable and available all day, permitting elderly individuals to participate at their convenience, without a set timetable. These clubs offer physical, cultural, and leisure activities and might feature training facilities. Significantly, these clubs are intended not just for older individuals but also to engage their families. The goal is to encourage community involvement. Services offered to elderly individuals are entirely free, whereas services for other family members (if applicable) may involve a charge. The notion is that seniors gain inclusive and free access to all services, demonstrating our dedication to their dignity and welfare (3).

##### 3.1.3.2 Eligibility criteria for elderly Care services

The institutionalization of elderly individuals is consistently regarded as the last resort. Strict admission standards for care homes have been implemented, with oversight managed by a dedicated committee at the Ministry. Admission is dependent upon Saudi citizenship, the absence of contagious or hazardous illnesses, and an evaluation of potential threats (3). Applicants must be at least sixty years old and demonstrate a level of functional decline or incapacity that prevents them from working or managing their daily affairs independently. It is permissible to accept someone under the age of sixty if social services prove the need to be included in the home services. New provisions within the Elderly Rights Law further stipulate that no senior individual can be placed in an institution without their clear approval, and it explicitly states that the main duty for caregiving rests with the family. The law outlines a responsibility hierarchy in caregiving: first, the partner (if agreeable); second, the father or mother; third, the children; and fourth, brothers and sisters. Social work initiatives are in place to support families who feel unable to provide care due to financial, emotional, or physical limitations (3). These initiatives include comprehensive socio-economic evaluations, counseling for families, and assistance systems designed to enable families to persist in providing care, including financial aid to family caregivers when required, as specified in the legislation. Consequently, institutionalization occurs only when the person is unaware or unable to consent, a court order requires it, or the circumstances present a significant safety threat (3).

For home care services, recipients must have a referral from a physician, be a member of a group targeted by the program, and their health must be comparatively stable to qualify. Secondly, the geographical location of the recipients should fall within a 50-km radius (30-min drive) of the hospital, the transfer should have the approval of the head of the family, the home should be conducive and safe to provide curative care, and a family member to act as a caregiver should be present (46). Palliative Care (PC) patients are individuals who show evidence of terminally and incurably diseases, plus an active do-not-attempt-resuscitation (DNAR) order, have stopped having curative lines, and have accepted care from PC and their families. By bringing PC to the patient, HHC programs do serve to take some eligible patients off the waiting list. Nevertheless, the care is usually transferred out of home care and back to hospital-based care again once the home-care situation becomes unmanageable (47).

#### 3.1.4 Modernization of elderly care services in Saudi Arabia

The rapid growth of Saudi Arabia’s elderly population is a primary driver of the development of the elderly care sector. Significant investments are enhancing the elderly care infrastructure. MOH has prioritized long-term care and rehabilitation, with plans to introduce specialized elderly care services in four hospitals in 2023, offering in-home dialysis, ventilator, and palliative care (48). HRSD operates 12 senior residential care homes, providing free comprehensive care to vulnerable elderly individuals, though capacity remains limited, with 56.8% of users concentrated in the Makkah region (5). The amount of money raised by private sector providers like Baraya Extended Care in 2025, up to $124 million to increase long-term care facilities, is an indicator that there is a strong private investment (49). The establishment of community-based care centers and day care programs is also gaining traction, catering to elderly individuals seeking social engagement and non-residential support (39).

Digital health technologies provide the elderly care sector with an opportunity to transform and modernize in alignment with the goals set in Vision 2030. Such telemedicine-based systems as the Sehhaty application operated by the MOH have provided more than 8.6 million consultations within the reach of elderly patients, especially rural ones (50). Aging-in-place solutions are supported using wearable devices that track vital signs and send alerts in case of an emergency, such as the Me Kaaz NEDA Band (51). Diagnostic and medication compliance is improved with the help of artificial intelligence applications, which are supported by the Saudi Data and AI Authority (SDAIA). The condition of diabetic retinopathy falls into this category, and the problem affects 1 in every 5 patients with diabetes aged over 70 (52). Seha Virtual Hospital is currently offering a variety of services, such as teleconsultations and AI-based care facilitated by smart algorithms, providing Elderly and more than 400 k people with health care (53). Ms. Ibtisam Al-Humaizi shared that Saudi Arabia represented the Kingdom at the 12th annual international conference on elderly care in Sharjah, showcasing innovative models and the contributions of the non-profit sector. Kabeerna Azeez” (“Our Elder is Precious”) and “Taqdeeruh Muwajih” (“Respecting Him Face to Face”) have been launched. Another initiative is being prepared for the International Day of Older Persons (3). These technological advancements are complemented by broader policy and structural reforms that seek to strengthen the healthcare system’s capacity to meet the needs of the aging population.

The Kingdom’s mHealth landscape has also expanded rapidly. Alharthi (54) found that mHealth application use among Saudi older adults has grown substantially in recent years, yet a persistent digital divide remains: older adults in rural areas and those with lower educational attainment were significantly less likely to use mHealth tools, even when mobile infrastructure was present. A separate study by Alharthi (55) catalogued current mHealth features available for Saudi elderly users, noting that most applications focus narrowly on appointment booking and medication reminders while neglecting broader geriatric needs such as cognitive health monitoring, fall detection, and caregiver integration. These findings underscore that digital infrastructure expansion alone is insufficient; application design must be co-developed with elderly users and aligned with the full spectrum of geriatric health needs.

The 2021 Private Sector Participation Law aims to increase private sector involvement from 20 to 35% by 2030, targeting privatization of 290 hospitals and 2,300 primary health centers, including elderly care facilities (56). Vision 2030 plans to recruit 175,000 healthcare professionals by 2030, including 64,000 nurses and 42,000 allied health workers, to address elderly care shortages, with a 65% increase in Saudi professionals since 2016 (57). The MOH also emphasizes preventive and community-based care to reduce hospital reliance. The “doctor-for-every-family” model targets 20 million beneficiaries, including the elderly, by improving primary care access (48). To achieve a comprehensive quality of life for older people across all regions of the Kingdom, involving the development and expansion of new services and increased engagement from various sectors.

#### 3.1.5 Governance and legal oversight of the elderly care sector

In Saudi Arabia, the elderly care sector operates under a strong regulatory framework designed to ensure quality, accessibility, and patient safety, aligning with the objectives of Vision 2030. The law Social Welfare, administered by the HRSD, regulates the government-operated senior residential facilities where the elderly can access these facilities free of charge, especially those with functional dependencies or those who lack families (5).

The Basic Law of Governance is clear on the rights of the citizens and the families in old age under Article 27, stipulating that the state shall support the social insurance and promote generous participation (7). The notable revolution is the new Elderly Rights and Care Law that was approved by the Council of Ministers in 2022 and provides special privileges to protect the social, financial, and legal rights of the elderly. This legislation also entails strict charges, such as fines or jail terms, in case of financial abuse or exploitation of the seniors. Also, it orders the research and data gathering to create the services specific to the elderly, encourages the work of volunteers, and demands the adjustment of the public facilities, commercial premises, residential areas, and mosques to their needs (7).

Furthermore, the mechanism of the elderly care is explained in the Executive Regulations of this Law, and it is described in Article 23. The provisions of Article (2) gave the Ministry of Human Resources and Social Development the role of coordination in the work to provide a rights-preserving and dignified environment, community awareness, provision of statistical data, organization of skills-enhancing programs, and the activation of able seniors to continue working (7). Article (3) confirms the right of older adults to live with their families, who bear the primary responsibility for their care and accommodation.

These local initiatives are compatible with global values, most significantly a 1991 United Nations General Assembly document on elderly rights, which specifies five fundamental principles: Independence (access to basic needs, work, education), Participation (in policy- making, knowledge transfer, associations), Care (family and healthcare provision with a human rights focus), Self-Realization (developing potential through community resources), and Dignity (living securely, free of exploitation, with respect regardless of economic contribution). The domestic legal system, as well as the international standards, are both pursued to guarantee a full, dignified, and rights-guaranteed life to older individuals (7).

The major endorsement and licensing body of the Saudi elderly care facilities, such as nursing homes, rehabilitation centers, and home care providers, is the Saudi Central Board for Accreditation of Healthcare Institutions (CBAHI). The CBAHI standards, which are based on International Society for Quality in Health Care (ISQua) principles, address the safety of infrastructure, infection prevention, and the qualification of the staff (58). To be legally operational, elderly care facilities are required to undergo CBAHI accreditation, which is renewed every three years; compliance is checked during annual inspections. Medical devices in elderly care must be approved before sale and monitored once in the market by the Saudi Food and Drug Authority (SFDA) to retain their safety and efficacy (59).

The standards of quality in elderly care are implemented by the CBAHI National Standards of Long-Term Care that propound the requirements of evidence-based procedures, patient-centered provision, and constant quality improvement (60). These standards urge facilities to survey patient satisfaction regularly and provide clinical outcomes, which helps eliminate gaps in care quality that were observed in previous studies (61). MOH has focused on the standardization of the course of treatment to minimize older care delivery variability in the Healthcare Transformation Strategy (36). The 2019 Patient Bill of Rights by MOH also guarantees the rights of elderly patients regarding dignity, privacy, informed consent, and culturally competent care, with measures to prevent neglect and abuse, including compulsory reporting procedures (62). Wheelchair ramps and Braille signage are needed as accessibility accommodations, yet they are not evenly distributed, particularly in rural regions.

#### 3.1.6 Residents overview

The elderly care industry primarily encompasses residential care homes, home-based care services, and community-based support programs, with a significant focus on government-operated facilities under the HRSD. Drawing on recent studies and official data, the profile of residents has been examined.

##### 3.1.6.1 Demographics

The Kingdom’s total population is projected to reach 35.3 million by mid-2024, showing a 4.7% annual growth rate relative to 2023 (63). The population of Saudi Arabia significantly rose by 15.6% over 2013–2022, and the proportion of the old-age population rose by 0.6%. Population ageing may be explained by the decrease in birth and fertility rates, which were rather slow and steady, as well as a relatively fixed death rate (64). The aging index, which is the ratio of the number of persons aged 60 and above to every 100 persons below 15, has been rapidly increasing since 1980–1995 in when the ratio was 6, and is predicted to be 160 by 2050, which highlights the increasing percentage of older adults who need elderly care services (65). Within the elderly care sector, residents are mostly older, with studies signifying that the majority are aged 75 years and above. For instance, a cross-sectional study of 2,702 residents in senior residential care homes in 2022–2023 found that 59.8% were male and 40.2% female, with a mean age of 66.66 years. Riyadh and Qassim had the highest representation (9.8% each), and Tabuk had the lowest representation (5.2%) (66).

A 2024 study examining patterns and factors of senior residential care home use across the 13 administrative regions of Saudi Arabia found that 56.8% of older adults in the Makkah region utilized residential care homes, representing the highest concentration in the country. Most of the participants were males (67.8%), while females comprised 32.2%. The average age was 78.9 years (SD = 10.6), with women significantly older than men (*p* = 0.014). Illiteracy was common, with women being the most illiterate (82.4% compared to 69.3% in men; *p* = 0.006). Most of the participants were divorced (68.2%), and more men were divorced (84.9% of men and 33% of women) (5). Conversely, a retrospective study of 880 elderly individuals registered for home health care services at the Armed Forces Hospital in Southern Saudi Arabia in 2011 reported that 55.9% of residents were female (67). This suggests a potential variability in gender distribution, influenced by temporal, spatial, and institutional factors. Gender demographics across differing time periods and types of elderly care facilities indicate a complex interplay of socio-demographic elements that shape the elderly care landscape.

##### 3.1.6.2 Health status

Hypertension and diabetes mellitus are the leading morbidities in elderly care settings. A 2011 retrospective study of 880 elderly home care recipients in Southern Saudi Arabia reported hypertension prevalence at 59.1% and diabetes mellitus at 57.3% (67). These data are consistent with the Saudi National Survey on Elderly Health (SNSEH) 2006–2015, which reported a self-reported prevalence of 47% when including the use of anti-diabetic medication, much greater than in the general adult population (65). Stroke (34.9%), dementia (28.5%), osteoarthritis (24.2%), Alzheimer’s disease (21.4%), and osteoporosis (17.2%) are also prevalent, particularly among female residents, who exhibit a statistically significantly higher burden of multimorbidity (67). The 2018 Saudi Family Health Survey further noted that 85.91% of elderly respondents aged 60 and older suffered from at least one chronic illness, with 17.35% reporting poor self-perceived health (68).

Multimorbidity, defined as the presence of two or more chronic conditions, is a critical concern, with 89% of home care recipients in the 2011 study having two or more morbidities (67). The prevalence of multimorbidity in Saudi Arabia (approximately 50%) is comparable to global figures, such as 51% in Australia and 52.8% in the United Kingdom, but lower than the 71% reported in Kuwait (66, 69–71). These conditions contribute to reduced quality of life, with each additional chronic disease decreasing quality of life by 4.37% (72).

##### 3.1.6.3 Level of dependency

Dependency is closely linked to chronic conditions such as dementia and stroke, which impair activities of daily living (ADLs). A 2015 study in Jeddah found that elderly residents in elderly care settings had limited ADL capacity, necessitating caregiver support for tasks such as mobility and personal care (73). A major concern is the risk of falls, which was estimated at 4 percent in elderly people in primary care, but with a greater range (up to 49.9 percent) in other studies, which requires preventive measures in elderly care facilities (74). The HRSD’s Social Security Agency provides in-kind assistance, such as wheelchairs and hospital beds, to address these needs, particularly for home care recipients (66).

#### 3.1.7 Human resources in the Saudi elderly care sector

Nurses are the primary providers of geriatric care and are therefore well-positioned to serve as team leaders coordinating healthcare services for older adults (8). The elderly care workforce forms part of the broader healthcare workforce; however, data specific to elderly care remain limited and are not systematically reported. A ministry report indicates that the nursing workforce reached 235,461 in 2023 across both public and private sectors, although the proportion working specifically in elderly care settings (e.g., HRSD senior care homes and private nursing facilities) is not quantified (75). Overall, the nursing workforce increased by 23% between 2017 and 2024 (76), reflecting general health system expansion, while regional disparities persist, with nurse-to-population ratios ranging from 3.13 to 9.89 per 1,000 population.

In contrast, data on caregivers and allied health professionals remain limited in official statistics. Similarly, physicians working in elderly care constitute only a small proportion of the total physician workforce (122,356) (77). The HRSD’s residential care homes, which serve a relatively small number of older adults, operate with a limited workforce, and no publicly available data exist on exact staffing levels (66).

#### 3.1.8 Financing models for elderly care in Saudi Arabia

The financing of elderly care services in Saudi Arabia relies on a multifaceted framework that integrates government funding, private out-of-pocket payments (OOP), health insurance contributions, charitable donations, and private sector investments. Each source plays a distinct role in addressing the needs of the elderly population, shaped by the Kingdom’s cultural, economic, and policy contexts (78, 79).

##### 3.1.8.1 Financial sustainability and income streams in the elderly care sector

The Saudi government, via the MOH and HRSD, continues to be the main source of funding for elderly care services, especially for public institutions. In the national budget, healthcare and social development are prioritized, with the government designating $50.4 billion in 2023, which accounts for 16.96% of the overall budget (80). This funding supports a network of public hospitals, residential care homes, and primary healthcare centers, including 12 senior care homes operated by the HRSD, which provide free, comprehensive care encompassing health, social, and mental support (5). MOH also contributes indirectly by funding primary healthcare centers that offer geriatric services, though elderly care-specific allocations are less clearly delineated in public budgets (79). The reliance on oil revenues, which account for over 90% of government income, underpins this funding but raises concerns about long-term sustainability due to volatile oil markets (81).

Out-of-pocket payments constitute a significant source of funding for elderly care, particularly in private facilities such as nursing homes and home-based care services. These expenses are typically not covered by insurance and must be paid by the older adults or their families. These expenses can lead to terrible financial problems for low-income families, underlining the need for extended public or insurance-based assistance (78). The HRSD also tends to fund non-profit organizations where low-income seniors can receive free or subsidized services based on home care, assistive devices, and residential care (78).

In the absence of official statistics on state expenditure for LTC, it can be inferred that institutional LTC remains underdeveloped, given the limited number of LTC facilities- only 15- and the availability of just 1,567 beds for LTC recipients as of 2020. Saudi Arabia is also seeking to activate the private sector in line with Saudi Vision 2030 (64). LTC for the elderly poses a significant challenge for the Saudi Arabia due to a general shortage of healthcare workers, including formal LTC staff. This, combined with the low number of LTC facilities, means that patients with LTC needs occupy between 20.0% and 30.0% of beds in public hospitals, which is more financially burdensome for both LTC recipients and the state (64).

##### 3.1.8.2 Financial allocation between providers and service users

The Saudi government, through HRSD and MOH, fully funds public elderly care services, as mandated by Article 27 of the Basic Law of Governance, which ensures support for citizens in old age (7). Under the National Transformation Program (NTP), the government is funding five model oases for elderly care, which include health services, physiotherapy, and recreational facilities, all provided free to residents (7). The HRSD also supports 13 non-profit civil societies specializing in elderly services to ensure nationwide coverage, with funding allocated to operational costs (7). In 2022, expenditures by the government on health and social development amounted to SAR 393 billion (around 10% of GDP), encompassing these programs, although specific funding for elderly care is not specified (82). Private providers, such as hospitals and home healthcare services, charge for chronic disease management and rehabilitation, but their fee structures are not publicly available. The non-profit Saudi Elderly Support Organization, “WAQAR,” funds its relief and educational programs through government grants, donations, and sponsorships, supporting public efforts (7).

Under the Elderly Rights and Care Law (37), eligible older adults receive services free of charge, including medical equipment (e.g., wheelchairs, hearing aids) and funding to support their care and daily maintenance. The Elderly Privilege Card offers priority and discounts on all forms of public, private, and non-governmental services, thereby reducing the out-of-pocket expenses of Saudi citizens (37). The Council of Cooperative Health Insurance aims to increase the number of individuals with private health insurance to 21.7 million by 2030, which could help alleviate the pressure on private care costs; however, subsidies for elderly care are not elaborated upon (82).

## 4 Discussion

Thirty years of research on elderly care in Saudi Arabia, from the early Exploratory Phase documented by Chen et al. (19) to the current Synergistic Development Phase, yields a body of evidence that is richer in its descriptive dimensions than in its critical and comparative depth. This discussion synthesizes key challenges, draws comparative lessons from Gulf Cooperation Council (GCC), MENA regional, and international systems, and articulates the research agenda that the next three decades of scholarship must address if Saudi Arabia’s aging population is to be served equitably and effectively.

### 4.1 Key challenges facing elderly care in Saudi Arabia

Four studies analyzing Saudi Arabia’s elderly care system report that it faces intertwined challenges in resource capacity, caregiver support, cultural norms, and technology integration. One survey of healthcare providers notes that high costs, insufficient training, and management barriers impede the adoption of Ambient Assisted Living devices (83). A study of 315 informal caregivers reveals a moderate burden, with 78.1% reporting musculoskeletal issues, and many calling for increased access to medical devices and training (84). Another survey of 244 decision-makers emphasizes deficiencies in services and resources, while highlighting a strong preference for family-based care (85). In addition, a descriptive study among caregivers in Riyadh finds a lack of geriatric specialists, poor coordination, and low societal awareness regarding the needs of older women (86).

#### 4.1.1 Rising chronic disease burden and complex care needs

The Kingdom’s population is projected to reach 35.3 million by mid-2024, reflecting a 4.7% annual growth rate from 2023 (68). This presents a major challenge to elderly care; due to the distributional rising life expectancy and the high rates of non-communicable diseases such as diabetes (57.3 percent) and hypertension (59.1 percent) (87). Such population change spurs the need for specialists, long-term care, and 89 percent of older care residents are affected by multimorbidity, which puts a strain on the limited infrastructure, including 12 government-managed care homes with a capacity to serve only 415 beneficiaries (5). The workforce shortage exacerbates care delivery, as 57% of nurses are expatriates, and the turnover rate is high (20–30% annually), with limited geriatric training (8). The elderly have a low level of health literacy (95.8 percent of the women are illiterate), which will make it difficult for them to follow modern care models (88). The policies intended to address these issues are outlined in the Healthcare Transformation Program 2030, which aims to focus on privatization and digital health integration in healthcare challenges. However, rural disparities and gaps persist despite these regulations (36). Targeted training, a build-up of infrastructure, and culturally tolerant interventions are essential to age burden management.

#### 4.1.2 Financial constraints and sustainability challenges

In every healthcare system, a major challenge is how to generate sufficient resources, since a strong health sponsorship structure raises adequate funds so that people can access necessary services without financial worries (61). Despite extensive government financing, primarily supported by oil revenues, the healthcare system, including elderly care, faces increasing pressure due to a rapidly aging population, a rising rate of chronic illnesses, and escalating healthcare costs. A study was conducted to assess the financial sustainability of Saudi Arabia’s publicly funded healthcare system. The results indicated significant risks to the system’s financial viability. Revenues, gross domestic product (GDP), government budget, and the MOH budget in Saudi Arabia are all directly influenced by oil prices (56). A qualitative study examining householders’ attitudes toward healthcare financing in Saudi Arabia shows that citizens are inclined to contribute to a national health insurance system only if improvements are made to public healthcare quality. Conducted by Al-Hanawi et al., this study explored public opinion about the current healthcare system and preferences for a future national health insurance model (78).

The study’s findings indicate that while there’s dissatisfaction with current public healthcare, specifically regarding appointment availability, waiting times, and drug availability, there’s a notable openness to contributing financially to a system that addresses these concerns. Participants expressed varying preferences and expectations regarding the structure of a potential national health insurance system. Budget allocation notably favors hospital services over primary care, creating an imbalance (79). The proposed solutions involve utilizing intelligent technology to achieve better patient outcomes and operational efficiency, implementing value-based healthcare models in which reimbursement is dependent on health outcomes rather than service delivery, and increasing access to affordable healthcare by either expanding insurance coverage or maintaining the cost of medications (89). Public-Private Partnerships (PPPs) are seen as a vital mechanism to enhance the private sector’s share in healthcare delivery and increase infrastructure and funds, thus lessening the financial burden on the government and contributing to the sector’s long-term financial flexibility (79).

#### 4.1.3 Workforce challenge

Despite efforts to increase the number of Saudi healthcare graduates, the present output is not adequate to meet the growing demand and replace retiring or leaving professionals, particularly in the nursing field (90). The dependence on immigrant healthcare workers raises concerns about sustainability and potential disturbances due to visa or policy changes (91). The issue of low female participation in nursing is a concern, and this is largely because of the poor image associated with the profession, and even more so by religious and cultural barriers. Adoption of a health workforce development policy that incorporates local culture, values, and social ties is highly necessary (92). Although Saudi Arabia has invested in training and scholarships for the medical staff, it has been required to increase training programs, notably the training in specializations, such as family medicine. The MOH should be accorded more autonomy in areas of workforce development and recruitment (93). Other than that, the health workforce is struggling with numerical shortage, skill inequity, gender disparity, and access issues (94).

According to a study conducted in Jazan General Hospital, burnout incidence was found to be very high among nurses, where emotional exhaustion and depression were the most common among nurses working long hours. This may cause turnover and reduce the quality of care (89). Migrated caregivers also exhibit high turnover due to low wages, lack of job security, and limited career progression opportunities (95). While global workforce planning models offer potential solutions, Saudi Arabia lacks localized, data-driven research on workforce dynamics, recruitment challenges, and retention strategies (96). A 2023 media report highlighted instances of neglect in private nursing homes due to insufficient staffing, raising concerns about the quality of care for vulnerable elderly residents. Additionally, the push for Saudization introduces ethical tensions, as rapid localization may prioritize quotas over competence, potentially affecting care standards if training programs are not scaled appropriately (97). Addressing workforce challenges requires comprehensive workforce planning, structured training programs, and policies that promote healthcare worker retention (96).

#### 4.1.4 Technological adoption and digital literacy challenges

Lack of digital literacy among older citizens and elderly care professionals is a major impediment to the effective use of technology. A qualitative study published in Riyadh in 2025 identified digital literacy among older populations as a central barrier to telehealth adoption, alongside the low availability of appropriate devices (43). Despite 100% mobile-cellular network coverage, a persistent gap in affordability and usability remains, particularly among illiterate or low-educated elderly populations (98). Knowledge of telehealth systems among healthcare providers is also insufficient, with only 47% reportedly using telehealth, and technophobia and inadequate skills cited as key obstacles (99). Additionally, the lack of clearly defined digital literacy strategies targeting both older adults and caregivers further limits effective technology uptake (98).

The digital divide in Saudi Arabia’s elderly care context is multidimensional and extends beyond infrastructure availability. Three interrelated layers contribute to this divide. First, device access; despite near-universal mobile network coverage and 5G penetration exceeding 77% nationally and over 94% in Riyadh, older adults, particularly women and rural residents, disproportionately lack access to smartphones or tablets due to cost, unfamiliarity, or intra-household social dynamics (100, 101). Second, digital competency; even among those with access to devices, the ability to navigate health applications, manage login credentials, and interpret digital health information remains limited. Alharthi (54) found that mHealth adoption among older adults correlated strongly with educational attainment and prior digital exposure, independent of income or device availability, indicating that digital inclusion efforts must prioritize literacy, not just connectivity. Third, content appropriateness; existing mHealth applications in Saudi Arabia insufficiently address the specific needs of older users, focusing primarily on appointment scheduling rather than chronic disease management, fall prevention, or caregiver communication, areas more relevant to elderly individuals with multimorbidity (55).

Although digital infrastructure has advanced significantly, rural areas continue to experience inadequate internet service provision and limited access to devices due to affordability constraints. Furthermore, the high cost of establishing and maintaining health IT infrastructure—including advanced data encryption for security—poses challenges for smaller elderly care facilities (101). At the system level, the Ministry of Health (MOH) is in the process of establishing a National Health Information Exchange (HIE), but interoperability challenges persist, restricting coordinated and cross-regional care delivery for elderly populations (102). To address these gaps, the Ministry of Communications and Information Technology (MCIT) and MOH are expanding digital infrastructure through 5G networks and SDAIA-led artificial intelligence initiatives (100).

Comparatively, Saudi Arabia’s digital health trajectory diverges from other MENA contexts. Countries such as Iran and Turkey have implemented similarly ambitious national digital health platforms but have invested more extensively in geriatric-specific telehealth modules and structured digital literacy programs within primary care (28). Evidence from Israel’s digital health initiatives targeting its Arab minority population, sharing some cultural similarities with Saudi elderly populations, demonstrates that co-designed digital tools, delivered through trusted intermediaries such as community health workers, significantly improve uptake among older, low-literacy users (30). Saudi Arabia’s adoption of community-based telehealth hubs reflects a similar approach; however, implementation remains uneven and lacks systematic evaluation (43).

Addressing these challenges requires a coordinated, multi-level strategy. First, targeted digital literacy programs should be embedded within primary healthcare visits and community centers, using Arabic-language, pictographic, and voice-guided interfaces tailored to low-literacy elderly users. Second, mHealth applications should be subject to geriatric-specific usability standards, potentially integrated into CBAHI accreditation frameworks, which currently lack such regulatory requirements (58). Third, community health workers and family caregivers should be trained as digital intermediaries, similar to “health navigators” in Canadian and Scandinavian models, to bridge the gap between technology and elderly users (43, 98). Community-based telehealth hubs can further support this approach by providing hands-on training and culturally sensitive support. Through continued investment under Vision 2030 in digital health infrastructure, training, and culturally appropriate care models, Saudi Arabia can strengthen its elderly care sector to meet the demands of a growing aging population without compromising its cultural context.

### 4.2 What Saudi Arabia’s elderly care experience offers to the region and the world

Saudi Arabia’s legal architecture for elder care is among the most explicit in the MENA region. The 2022 Elderly Rights and Care Law’s provision of a statutory care responsibility hierarchy, placing the spouse first, followed by parents, children, and siblings, and its requirement of informed consent for institutionalization are normative innovations that other GCC and Arab states have not yet enacted. Jordan, for example, continues to rely on social welfare frameworks that provide no equivalent legal protection against non-consensual institutionalization (28). Other GCC and Arab states seeking to modernize elder care policy could draw directly on the Saudi legal model as a template.

Second, Saudi Arabia’s experience with the cultural stigma around institutional care and its gradual, socially differentiated erosion offers a transferable lesson: stigma reduction is not achieved through information campaigns alone but through the redesign of care services to be culturally congruent. The Saudi model of social clubs that include family members, day-care centers aligned with working-day schedules, and the emphasis on the ‘Kabeerna Azeez’ (Our Elder is Precious) narrative campaign represent a deliberate strategy of stigma-sensitive institutional design that other Muslim-majority societies could adapt (3, 34).

Third, Saudi Arabia’s rapid oscillation between very limited elderly care infrastructure and ambitious Vision 2030 targets, from 15 LTC facilities in 2020 to a projected multi-billion-dollar market by 2033, illustrates both the potential and the risk of policy-driven rapid scale-up (41, 64). Countries in the early stages of elder care system development, including several Sub-Saharan African nations with rapidly aging urban populations, may find in the Saudi trajectory both an inspirational precedent and a cautionary tale: rapid scale-up without workforce development and quality regulation risks producing quantity without quality.

Fourth, and perhaps most distinctively, Saudi Arabia’s religious and cultural context produces a care ethics that differs meaningfully from both Western liberal models (which emphasize individual autonomy) and East Asian Confucian models (which emphasize filial obligation without statutory backing). The Saudi synthesis, in which Islamic principles of family duty are reinforced by, rather than replaced by, state law, represents a third model of care ethics that has not been adequately theorized in the international gerontology literature and merits further scholarly attention (7, 32, 34).

### 4.3 Research gaps, overlooked populations, and methodological limitations

A critical audit of the studies reviewed reveals systematic patterns of omission that must be addressed in the next generation of elderly care research in Saudi Arabia.

The most striking gap is the near-total absence of data on elderly women outside of institutional settings. Of the studies reviewed, the large majority that examined gender either reported aggregate statistics or focused on male-dominated residential care home populations, where men outnumber women by approximately two to one (5, 66). Yet women constitute the majority of informal caregivers, are more likely to be widowed and thus socially isolated in later life, and face the highest rates of illiteracy and lowest rates of pension coverage among Saudi elderly (5, 88). This intersection of gender, aging, and social vulnerability is critically under-researched. Future studies should prioritize community-based samples with overrepresentation of older women, particularly in rural governorates.

A second major gap is the lack of longitudinal research. The overwhelming majority of studies in this review are cross-sectional, meaning they capture a single point in time. This makes it impossible to trace care trajectories, assess the long-term outcomes of interventions, or model the relationship between socioeconomic change and care need over time. The HRSD’s residential care homes collect longitudinal administrative data that have not, to the authors’ knowledge, been systematically analyzed and published. Enabling researcher access to de-identified administrative datasets would represent a high-impact, low-cost research investment.

Third, non-Saudi elderly residents, primarily long-term expatriate workers who have aged in the Kingdom without access to the social care system, constitute an invisible population in the literature. Saudi law restricts residential care homes to Saudi nationals, creating a gap for a potentially substantial population of aging non-nationals whose care needs, legal status, and social support networks have not been studied (3, 7).

Fourth, the review reveals an almost complete absence of health economic analyses specific to elderly care. There are no published cost-effectiveness studies comparing residential, home-based, and community care modalities in the Saudi context; no published analyses of the fiscal impact of the 2022 Elderly Rights Law; and no modeling of the long-term financial liability associated with the projected demographic transition. Without this evidence base, policymakers are making investment decisions of enormous magnitude without actuarial grounding.

Finally, qualitative research on the subjective experience of aging in Saudi Arabia is remarkably thin. Karlin et al. (34) remains the most cited qualitative study on Saudi older adults’ own perspectives on aging and care, yet it was conducted a decade ago in a context that has changed substantially. Ethnographic and narrative methods that give voice to older Saudis, including their preferences, fears, aspirations, and experiences of the formal care system, are essential to ensure that policy and service design are genuinely person-centered rather than paternalistic.

## 5 Conclusion

This systematic review demonstrates that Saudi Arabia’s elderly care system is at a critical juncture: while Vision 2030 has catalyzed meaningful reform—including expanded long-term care infrastructure, digital health integration, and the landmark 2022 Elderly Rights and Care Law—persistent structural challenges remain. These include workforce shortages, heavy dependence on migrant caregivers, cultural resistance to institutional care, rural service disparities, and a growing NCD burden among older adults. The way forward requires three strategic priorities: (1) targeted workforce development with a focus on geriatric specialization and Saudization without compromising care quality; (2) technology-supported, culturally sensitive care models that address digital literacy barriers; and (3) sustained investment in longitudinal research and health economic analysis to ground policy in evidence. Addressing significant data gaps on elderly care services, workforce composition, and gender-specific aging outcomes will be essential to support equitable and informed long-term planning.

Overall, thirty years of research on elderly care in Saudi Arabia have produced a valuable but incomplete body of knowledge. The descriptive picture is increasingly clear with a demographically pressured, culturally complex system caught between rapid policy ambition and structural constraints. The research must deliver an analytical and comparative deepening of this picture, including rigorous longitudinal data, health economic evidence, qualitative voice-centered studies, and comparative analyses that position Saudi Arabia within MENA and global gerontological discourse.

## Data Availability

The original contributions presented in the study are included in the article/supplementary material, further inquiries can be directed to the corresponding author/s.
